# Transcriptome profiling of whisker follicles in methamphetamine self-administered rats

**DOI:** 10.1038/s41598-018-29772-1

**Published:** 2018-07-30

**Authors:** Sang-Hoon Song, Won-Jun Jang, Jihye Hwang, Byoungduck Park, Jung-Hee Jang, Young-Ho Seo, Chae Ha Yang, Sooyeun Lee, Chul-Ho Jeong

**Affiliations:** 1 College of Pharmacy, Keimyung University, Daegu, 42601 Republic of Korea; 2 School of Medicine, Keimyung University, Daegu, 42601 Republic of Korea; 3 College of Oriental Medicine, Daegu Hanny University, Daegu, 42158 Republic of Korea

## Abstract

Methamphetamine (MA) is a highly addictive psychostimulant that disturbs the central nervous system; therefore, diagnosis of MA addiction is important in clinical and forensic toxicology. In this study, a MA self-administration rat model was used to illustrate the gene expression profiling of the rewarding effect caused by MA. RNA-sequencing was performed to examine changes in gene expression in rat whisker follicles collected before self-administration, after MA self-administration, and after withdrawal sessions. We identified six distinct groups of genes, with statistically significant expression patterns. By constructing the functional association network of these genes and performing the subsequent topological analysis, we identified 43 genes, which have the potential to regulate MA reward and addiction. The gene pathways were then analysed using the Reactome and Knowledgebase for Addiction-Related Gene database, and it was found that genes and pathways associated with Alzheimer’s disease and the heparan sulfate biosynthesis were enriched in MA self-administration rats. The findings suggest that changes of the genes identified in rat whisker follicles may be useful indicators of the rewarding effect of MA. Further studies are needed to provide a comprehensive understanding of MA addiction.

## Introduction

Addiction is a representative disorder of the brain’s reward system and occurs over time from high chronic exposure to an addictive stimulus, such as an abuse drug^1^. The defining feature of addiction is compulsive and out-of-control drug use^2^. The uncontrolled drug use may lead to criminal or anti-social behaviour, violence, physical dependence, or psychological addiction^3^.

Methamphetamine (MA), the second most abused illegal psychostimulant, affects the central nervous system^4^. MA has pervasive effects not only on the dopaminergic system, but also on diverse neurotransmitter systems, such as noradrenergic and serotonergic systems, in the brain. Converging evidence suggests that chronic MA use is associated with immunological dysfunction and impaired neurogenesis in the limbic dopaminergic system^5^. The reported neurotoxicity induced by MA might be related to reactive oxygen species^6^, apoptotic processes^7^, and excitotoxicity^8^, which might be a major cause of neurodegenerative disorders, in the dopaminergic and serotonergic systems. Clinical observation suggests that MA causes long-lasting injury to the brain. Moreover, diverse clinical symptoms, such as depression, suicidal ideation, and psychotic behaviour, are observed in people who are addicted to MA^9^.

MA abuse and addiction have become a serious worldwide issue since the 1990s. Ease of manufacture and distribution is the major factor linked to increased use of MA^10^. In Republic of Korea, MA is the most widely used illegal drug, and 100% of persons treated for drug problems in the country are MA abusers^11^. In particular, MA abuse is a growing serious problem in youths; thus, investigation on MA abuse is needed to understand the seamy side of MA. Therefore, testing for MA abuse is crucial for assessing the reality of intoxication and evaluating the level of drug impairment.

Testing for MA abuse is largely dependent on biofluids, such as urine, blood, and plasma. Urine is the most used traditional specimen; urine testing is a valuable measure of drug use in patients who use the drug regularly^12^. However, urine testing has limitations for those with moderate drug use because of the short drug detection time^13^. In contrast, the use of the hair has several advantages, such as ease of collection, noninvasiveness, and a long detection window (months to years), for toxicological analysis^14^. Interestingly, a recent report suggests that scalp hair follicles can be used to find genes associated with brain diseases because the brain is an ectodermal tissue and shares the developmental origins with scalp hair follicle^15^. Moreover, the hair follicle system, which is a well-coordinated miniorgan within the skin, has been used to detect stressors, such as neurohormone, neurotransmitters, and cytokines^16^, as well as to diagnose traumatic brain injury^17^. Therefore, it could be valuable to study changes in gene expression in hair follicles, instead of brain tissues, in order to identify novel indicators related to the rewarding effect of MA in living donors.

The development of addiction is accompanied by changes in the signal transduction pathway and cellular gene expression in the brain. Many addiction-related genes and pathways have been identified^18–20^; however, the exact mechanism underlying MA addiction has not been fully elucidated. Previous studies have implied that exposure to MA causes complex molecular alterations; therefore, a comprehensive multigene level approach is needed to understand the drug addiction mechanisms, and it is important to identify the principal molecular network underlying MA addiction by analysing alteration in gene networks.

RNA-sequencing (RNA-seq) is transcriptome profiling and uses deep-sequencing technologies. RNA-seq is used to investigate the expression of unknown and known candidate genes with higher sensitivity than microarray. Genes that are significantly upregulated or downregulated can be determined in certain conditions by quantifying changes in RNA expression between different conditions^21^. However, RNA-seq alone may not provide pragmatic results in a multigenic disease, such as drug addiction, because of extensive raw data; therefore, data management and computational screening are needed. The integration of bioinformatics and biological network analysis enables RNA-seq to define specific groups of genes that are related to drug addiction behaviour^22^. Recent advances in bioinformatics facilitate analysis of complex signalling transduction in cells through investigation of multiple types of interaction networks^23^. Biological network analysis is a powerful tool using computable networks in the biological system^24^. Gene-gene association network analysis provides a novel method of analyzing a large number of differentially expressed genes by classifying them into different gene groups based on their function or signalling pathway annotation^25^. In the network analysis, degree, which is the number of edges connecting a node, and betweenness centrality (BC), which is how central the node is to the network, are used to show the importance of the genes^26^.

In this study, RNA-seq was used to illustrate transcriptome profiling of whisker follicles in MA self-administered rats. We also investigated alteration in the gene networks (rather than individual gene expression), signalling networks, and the signalling pathways, and the alteration may be responsible for the rewarding effect of MA. The results may serve to reveal the complicated gene-gene interaction mechanisms of the rewarding effect caused by MA.

## Results

### A MA self-administration rat model

**Figure 1:**
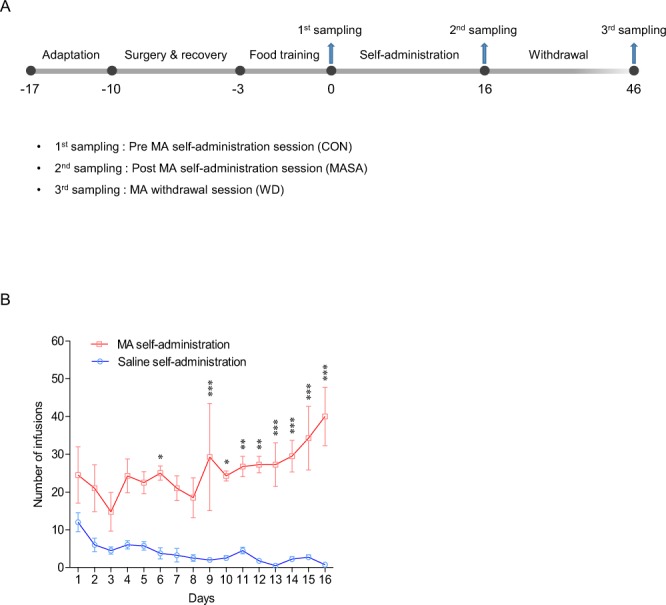
Methamphetamine self-administration (MASA). (**A**) The experimental timeline of saline or methamphetamine self-administration and the sampling time-points of MASA. (**B**) The number of infusions in saline or methamphetamine self-administration rats. MA self-administered rats (n = 6) had a significantly greater infusion number than did saline self-administration rats (n = 6). Statistical analysis was performed using the two-way analysis of variance (ANOVA) and the student Newman-Keuls test. Error bars represent the mean ± SEM (n = 6). *p < 0.05, **p < 0.01, and ***p < 0.001.

Saline or MA self-administration in rats was performed according to the experimental schedule as indicated in Fig. 1A. Figure 1B shows the number of lever responses during self-administration (2 h each day, 16 days) under the fixed-rate 1 (FR1) schedule. The number of drug infusions in MA self-administration rats gradually increased compared with that in saline self-administration rats and showed <10% variation during the last 3 days of the experiment. The mean number (mean ± SEM) of MA infusions on the final day was 40 ± 7.7. Subsequently, stably responding MA self-administration rats (n = 6) underwent a withdrawal session of 30 days. A two-way analysis of variance (ANOVA) showed a significant difference in the number of infusions between saline and MA self-administered rats (*p* < 0.01).

### RNA-seq and gene expression profiling

RNA-seq was performed using the whisker follicle samples collected from 3 representative rats before self-administration (CON), after MA self-administration (MASA), and after withdrawal (WD) sessions, and the three rats were selected based on the statistical procedure and showed less variation per day during the self-administration session. A total of 8,680 differentially expressed transcripts were analysed using one-way ANOVA in both groups. The quantitative value of each transcript abundance was used to perform a principal component analysis (PCA).

**Figure 2:**
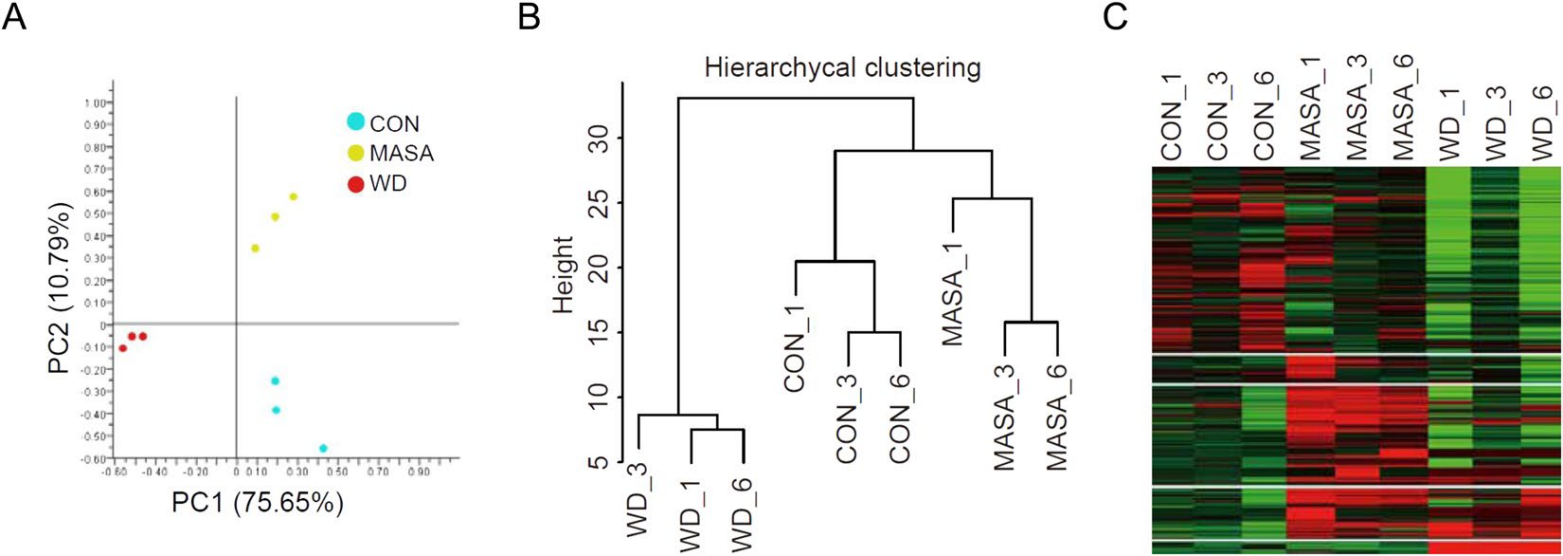
Quality assessment of RNA-sequencing (RNA-seq) data. (**A**) Grouping of samples *via* principal component analysis based on gene expression abundance. (**B**) Hierarchical clustering of genes. (**C**) A heat-map of the 8,680 expressed genes. Gene expression abundance decreases from red to green.

The differentially expressed genes at the three time-points (CON, MASA, and WD) were clustered based on the quantitative results of transcripts following the PCA analysis (Fig. 2A). The differentiation was primarily represented by the first principal component (PC1), accounting for 75.65% of the observed variance. The secondary principal component (PC2) showed 10.79% of the variance. The fundamental difference in RNA abundance between the three time-points accounted for the separation; therefore, principal components could achieve clear separation (Fig. 2A). In the hierarchical clustering analysis (Fig. 2B), it was found that the individual samples were well clustered according to their conditions. Distinct gene expression abundances at each time-point are presented as a heat map in Fig. 2C.

### Gene expression pattern analysis

**Figure 3:**
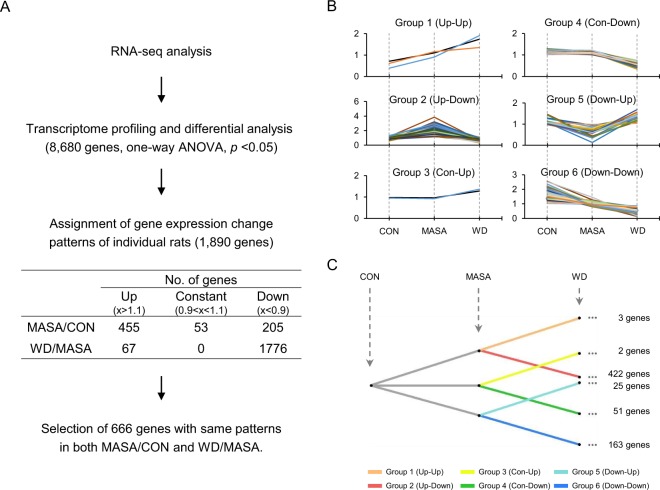
Gene expression pattern analysis of RNA-seq data. (**A**) Flow chart of RNA-seq data processing. Transcriptome profiling in whisker follicle samples collected at the three time-points (control [CON], MASA, and withdrawal [WD]). Differentially expressed transcripts among the three time-points were identified through a one-way ANOVA. (**B**) Gene expression pattern analysis. The expression patterns of genes were classified into six groups. All graphs are expressed as the normalised value of gene expression. (**C**) The number of genes in each group.

A total of 1,890 genes were identified with same expression patterns after primary data alignment and further filtration (Fig. 3A). In addition, 455 and 205 genes were up- and down-regulated, respectively, at the MASA session compared with the CON session (MASA/CON); 67 and 1,776 genes were significantly up- and down-regulated, respectively, at the WD session compared with the MASA session (WD/MASA) (Fig. 3A). It is noteworthy that more genes (1,776) were down-regulated after MA withdrawal. A time-series correlation of changes in expression identified 666 genes with differential expression patterns in the three rats. According to the mRNA abundance ratios (MASA/CON, WD/MASA), the genes were classified into 6 groups: 3 up-up regulated (Group 1), 422 up-down regulated (Group 2), 2 constant-up regulated (Group 3), 51 constant-down regulated (Group 4), 25 down-up regulated (Group 5), and 163 down-down regulated genes (Group 6) (Fig. 3B,C). Notably, the expression of 422 transcripts in Group 2 gradually increased at the MASA session and decreased to the baseline at the WD session (Fig. 3B,C). In contrast, expression of 163 transcripts in Group 6 decreased at the MASA session and then did not decrease further at the WD session (Fig. 3B,C).

### Network analysis of differentially expressed genes (DEGs)

**Figure 4:**
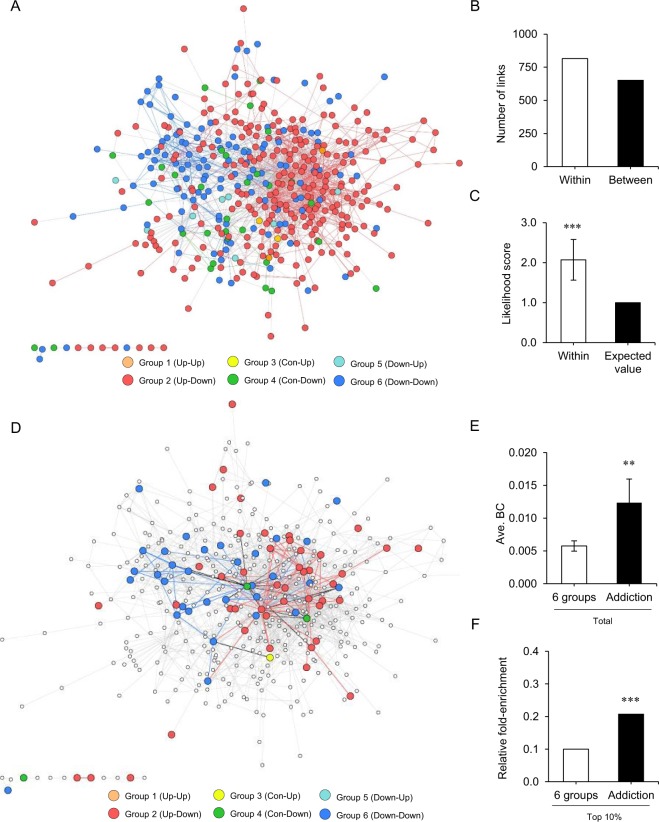
Functional association network analysis of differentially expressed genes. (**A**) Functional association network in MASA and WD rat whisker follicles. Nodes in the same group are coded with the same colour. Orange and red nodes represent up-regulation in MASA and up-down-regulation in WD, respectively. Yellow and green nodes show constant expression in MASA and up-down-regulation in WD, respectively. Cyan and blue nodes show downregulation in MASA and up-down-regulation in WD, respectively. The links between the same colour nodes are displayed using identical colours. (**B**) A comparison of link numbers “within” and “between” groups. The number of links according to link type. “Within” is a link to the identical group. “Between” is a link to the different groups. (**C**) Likelihood scores of “within” groups. Statistical analysis was performed using the Mann-Whitney U test. ***P < 0.001. (**D**) Distribution of addiction-related genes in the network. Links between the same colour nodes are displayed with identical colours. (**E**) Mean betweenness centrality (BC) scores of the six groups and addiction-related gene group (addiction). Statistical analysis was performed using the Mann-Whitney U test. **p < 0.01. (**F**) Relative fold-enrichment BC scores of the six groups and addiction-related gene group. Statistical analysis was performed using the hypergeometric test. ***p < 0.001.

Physical and functional links of altered genes were investigated based on the assumption that gene pairs that interact or share similar functions tend to interact within the cellular systems^27,28^. To analyse the changes in mRNA abundances under MA addiction and withdrawal, the functional association network was generated using the 432 seed genes among 666 altered genes based on information about the physical and functional links of these genes. The physical link represents protein-protein interaction, and the functional link represents a relationship between two proteins if they share a substrate in a metabolic pathway and are co-expressed, co-regulated, or involved in the same protein complex. It was found that the network was divided into two prominent subnetworks of up-down regulated (Group 2/red) and down-down regulated genes (Group 6/blue) based on gene expression patterns (Fig. 4A). Interestingly, more links were found within a group (link to the identical group) than between groups (link to the different group). Among 1,466 links in the network, 815 (55.6%) were found to be intra-regulatory links (Fig. 4B). Subsequently, the fraction of links per gene was measured. Links within a group were 2.07-fold more than the expected value (Mann-Whitney U test, *p* value = 2.59 e^−8^; Fig. 4C). The results indicate that the two distinct sub-networks of up-down regulated (Group 2) and down-down regulated (Group 6) genes tend to cluster themselves and might have distinct functional roles in the gene functional network.

### Network mapping of addiction-related genes

The gene functional network was analysed based on a topological parameter, BC, which reflects the importance of genes in the network. The BC score is the minimum number of links connecting one protein to another in the network; thus, a high BC score implies that genes undergo more number of the shortest paths in the network. Therefore, the protein with the highest BC score derives a lot of importance from its position in the network. To check whether the constructed network contains the gene sets involved in MA addiction, known addiction-related genes from Knowledgebase for Addiction-Related Gene (KARG) database were mapped to the functional network. Subsequently, 1,256 addiction-related genes were collected from 105, 346, 474, and 331 genes mapped from low-throughput (LT) data, mouse, human, and high-throughput (proteomics and microarray) data, respectively, in KARG. As a result, 82 addiction-related genes (6.5%) were mapped in the functional association network that consisted of 432 genes (Fig. 4D). The average BC score of known addiction-related genes was higher than that of other genes (Mann-Whitney U test, *p* = 0.006; Fig. 4E). The relative fold-enrichment of known addiction-related genes with the top 10% of BC scores was 2.07-fold higher than that of all genes in the 6 groups (Hypergeometric test, *p* = 5.38 × 10^−4^; Fig. 4F). Finally, top 43 ranked genes, by the BC score, were selected as possible markers of MA addiction (Table 1). *Hsp90ab1* (BC score, 0.2127), *Akt1* (BC score, 0.1694), and *Src* (BC score, 0.1171) were identified with the highest BC scores and might act as molecular bridges to connect multiple other proteins in this network. These genes have been previously reported as addiction-related genes^18,29,30^. Genes with high BC score tend to interact with many different functional groups and are important for controlling information flow in the network. Furthermore, 17 out of 43 genes (39.5%) were mapped as pre-designated addiction-related genes, suggesting that the current approach of network analysis confers a high level of reliability and confidence in the selection of genes that are related to MA addiction. Addiction-related genes with high BC scores could be identified based on their importance in information flow in the altered gene-gene network under addiction-withdrawal conditions.

**Table 1:** List of genes with top 10% betweenness centrality (BC) scores in the network.

Rank	Genes	Genbank	BC	Addic Re.	Group	Description	Normalised	
							MASA	WD
1	Hsp90ab1	NM_001004082	0.2127	O	G4	Heat shock protein 90 alpha (cytosolic), class B member 1 (Hsp90ab1)	0.94	0.51
2	Akt1	NM_033230	0.1694	O	G2	V-akt murine thymoma viral oncogene homolog 1 (Akt1)	1.58	0.80
3	Src	NM_031977	0.1171	O	G2	SRC proto-oncogene, non-receptor tyrosine kinase (Src)	1.31	0.66
4	Ranbp2	NM_001191604	0.0671		G5	RAN binding protein 2 (Ranbp2)	0.77	1.39
5	Mapk14	NM_031020	0.0586		G4	Mitogen activated protein kinase 14 (Mapk14)	0.96	0.58
6	Egfr	NM_031507	0.0568		G2	Epidermal growth factor receptor (Egfr)	1.30	0.67
7	Prkacb	XM_006233452	0.0447	O	G2	Protein kinase, cAMP dependent, catalytic, beta (Prkacb), transcript variant X2	1.23	0.65
8	Gnb2l1	NM_130734	0.0436		G6	Guanine nucleotide binding protein (G protein), beta polypeptide 2 like 1 (Gnb2l1)	0.78	0.45
9	Cav1	NM_031556	0.0419		G2	Caveolin 1, caveolae protein (Cav1), transcript variant 1	3.53	0.52
10	Cdh1	NM_031334	0.0385	O	G4	Cadherin 1 (Cdh1)	1.04	0.50
11	Cct7	NM_001106603	0.0363		G6	Chaperonin containing Tcp1, subunit 7 (eta) (Cct7)	0.85	0.54
12	Itgb1	NM_017022	0.0322		G2	Integrin, beta 1 (Itgb1)	2.02	1.16
13	Ppp1ca	NM_031527	0.0319	O	G6	Protein phosphatase 1, catalytic subunit, alpha isozyme (Ppp1ca)	0.81	0.50
14	Actr1a	NM_001106364	0.0313		G4	ARP1 actin-related protein 1 homolog A, centractin alpha (yeast) (Actr1a)	0.99	0.51
15	Tgfb1	NM_021578	0.0313	O	G2	Transforming growth factor, beta 1 (Tgfb1)	1.75	0.34
16	Sec61a1	NM_199256	0.0290	O	G6	Sec61 alpha 1 subunit (S. cerevisiae) (Sec61a1)	0.73	0.31
17	Cdk16	NM_031077	0.0274		G6	Cyclin-dependent kinase 16 (Cdk16), transcript variant 2	0.85	0.51
18	Ywhab	NM_019377	0.0262	O	G6	Tyrosine 3-monooxygenase/tryptophan 5-monooxygenase activation protein, beta (Ywhab)	0.76	0.52
19	Sdc2	NM_013082	0.0250		G2	Syndecan 2 (Sdc2)	1.77	0.67
20	Psmd2	NM_001031639	0.0236		G6	Proteasome (prosome, macropain) 26S subunit, non-ATPase, 2 (Psmd2)	0.77	0.45
21	Jak1	NM_053466	0.0232		G2	Janus kinase 1 (Jak1)	1.83	1.05
22	Rela	NM_199267	0.0232		G4	V-rel avian reticuloendotheliosis viral oncogene homolog A (Rela)	1.04	0.56
23	Vcp	NM_053864	0.0218	O	G6	Valosin-containing protein (Vcp)	0.78	0.51
24	Srebf2	NM_001033694	0.0211		G4	Sterol regulatory element binding transcription factor 2 (Srebf2)	0.94	0.55
25	Atp5a1	NM_023093	0.0206		G6	ATP synthase, H+ transporting, mitochondrial F1 complex, alpha subunit 1, cardiac muscle (Atp5a1)	0.79	0.44
26	Nrp1	NM_145098	0.0201	O	G2	Neuropilin 1 (Nrp1)	1.87	0.63
27	App	NM_019288	0.0197	O	G2	Amyloid beta (Aβ) precursor protein (App)	2.71	0.83
28	Eef1g	NM_001004223	0.0197	O	G6	Eukaryotic translation elongation factor 1 gamma (Eef1g)	0.77	0.33
29	Stat5a	NM_017064	0.0191		G2	Signal transducer and activator of Transcription 5A (Stat5a)	2.18	0.64
30	Itch	NM_001005887	0.0187		G2	Itchy E3 ubiquitin protein ligase (Itch)	1.22	0.87
31	Atp1a1	NM_012504	0.0183		G2	ATPase, Na+/K+ transporting, alpha 1 polypeptide (Atp1a1)	1.80	0.63
32	Zmiz1	NM_001108393	0.0176		G2	Zinc finger, MIZ-type containing 1 (Zmiz1)	1.72	0.68
33	Gli1	NM_001191910	0.0161		G2	GLI family zinc finger 1 (Gli1)	3.78	1.42
34	Prkg2	NM_013012	0.0158	O	G6	Protein kinase, cGMP-dependent, type II (Prkg2)	0.51	0.09
35	Ctbp2	NM_053335	0.0153		G2	C-terminal binding protein 2 (Ctbp2)	1.24	0.92
36	Hadhb	NM_133618	0.0147	O	G6	Hydroxyacyl-CoA dehydrogenase/3-ketoacyl-CoA thiolase/enoyl-CoA hydratase (trifunctional protein), beta subunit (Hadhb)	0.69	0.48
37	Nfkb2	NM_001008349	0.0144		G2	Nuclear factor of kappa light polypeptide gene enhancer in B-cells 2, p49/p100 (Nfkb2)	1.57	0.85
38	Stk11	NM_001108069	0.0143		G6	Serine/threonine kinase 11 (Stk11)	0.82	0.43
39	Vcl	NM_001107248	0.0141		G2	Vinculin (Vcl)	1.73	0.73
40	Pip4k2b	NM_053550	0.0136	O	G2	Phosphatidylinositol-5-phosphate 4-kinase, type II, beta (Pip4k2b)	1.46	0.74
41	Tle3	NM_053400	0.0134	O	G6	Transducin-like enhancer of split 3 (Tle3)	0.70	0.35
42	Map3k3	NM_001107058	0.0133		G2	Mitogen activated protein kinase (Map3k3)	1.41	0.72
43	Fbxw11	NM_001106993	0.0129		G2	F-box and WD repeat domain containing 11 (Fbxw11)	1.16	0.68

### Functional analysis using reactome

**Figure 5:**
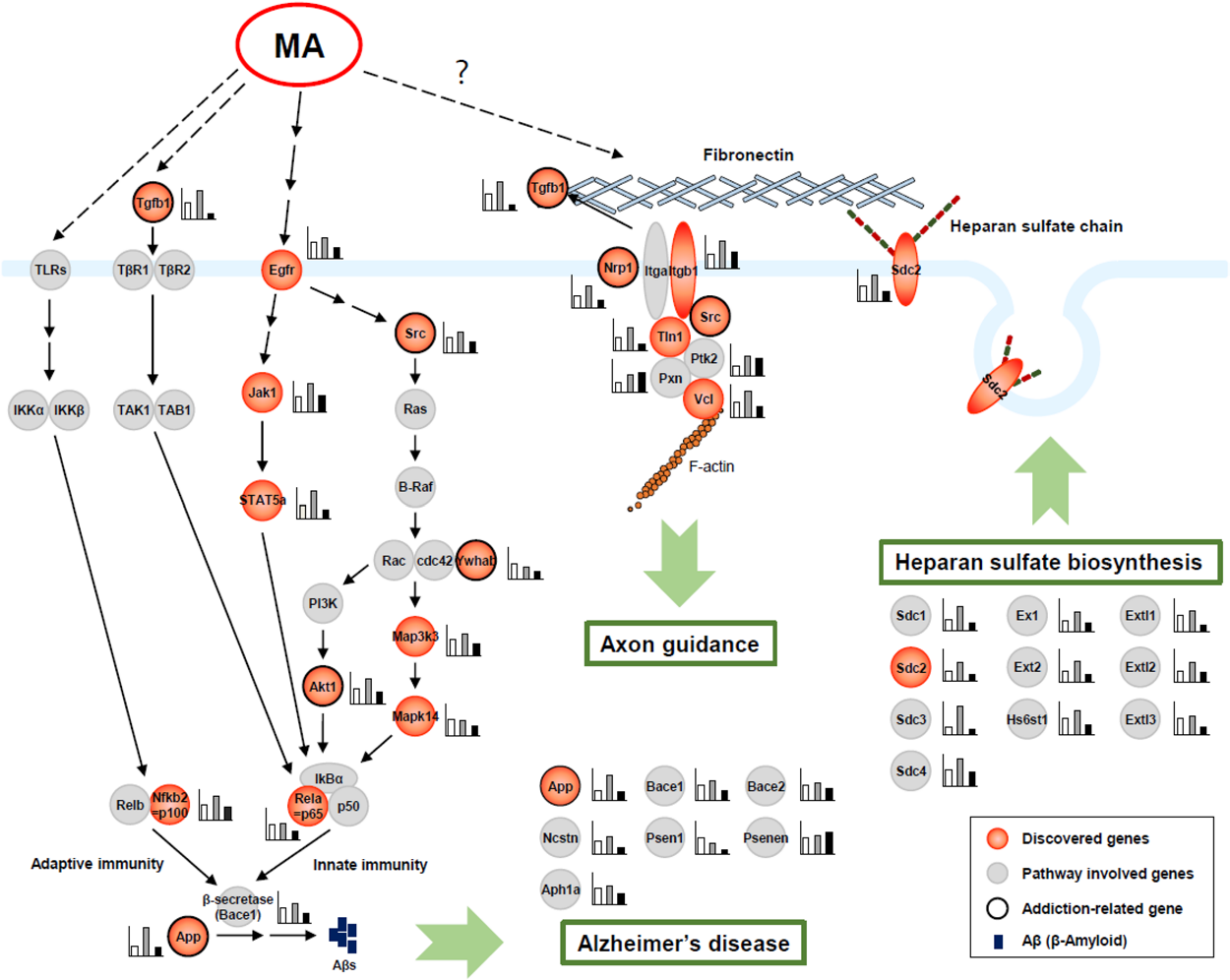
Schematic of the signalling pathways altered in MA self-administration. An increased expression of genes related to Alzheimer’s disease (AD) and heparan sulfate biosynthesis is observed in MASA. Activation of nuclear factor-κB leads to up-regulation of amyloid-β precursor protein, which is associated with AD.

To assess the biological relevance of the 43 top ranked genes, functional enrichment analysis was performed using the Reactome database (Reactome. 2004). Biologically related gene sets were identified and labeled with the Reactome terms. The main enriched pathways and genes are presented in Table 2. As expected, the pathways included genes that function in the regulation of cell-cell communication, developmental biology, diseases, extracellular matrix organization, hemostasis, the immune system, metabolism, and signal transduction. In addition, several genes associated with the progression of Alzheimer’s disease (AD) were observed (Fig. 5). It is noteworthy that all well-known signalling pathway-related genes, such as transforming growth factor beta 1 (*Tgfb1*), epidermal growth factor receptor (*Egfr*), and proto-oncogene tyrosine-protein kinase (*Src)*, activate nuclear factor-κB (NF-κB) and thereby regulate the expression of amyloid-β precursor protein (APP), which is associated with AD. Moreover, most genes associated with biosynthesis of heparan sulfate, a member of the glycosaminoglycan family, were upregulated at the MASA session (Fig. 5).

**Table 2:** Biological pathways and genes regulated by MA self-administration.

Pathways	**Genes**	***p*** **-Value**
**Cell-cell communication**		
Cell junction organization	Vcl	0.02
Cell-cell junction organization		
Adherens junctions interactions	Vcl	0.01
**Development biology**		
Axon guidance		
L1CAM interactions		
CHL1 interactions	Nrp1, Itgb1	0.01
**Disease**		
Diseases of signalling transduction		
Signalling by TGF-beta Receptor Complex in Cancer		
Loss of function of TGRBR1 in Cancer		
TGFBR1 LBD Mutants in Cancer	Tgfb1	0.05
Loss of function of TGRBR2 in Cancer		
TGFBR2 MSI Frameshift Mutants in Cancer	Tgfb1	0.03
Signalling by WNT in cancer		
TCF7L2 mutants-unbinded CTBP	Ctbp2	0.04
Oncogenic MAPK signalling		
Signalling by high-kinase activity BRAF mutants	Src, Ywhab, and Vcl	0.03
Signalling by moderate kinase activity BRAF mutants	Src, Ywhab, and Vcl	0.05
Paradoxical activation of RAF signalling by kinase inactive BRAF	Src, Ywhab, and Vcl	0.05
Diseases of Immune System		
Diseases associated with the TLR signalling cascade		
IKBKG deficiency-caused anhidrotic ectodermal dysplasia with immunodeficiency (EDA-ID) (via TLR)	Nfkb2, Rela	0.01
IkBA variant leads to EDA-ID	Nfkb2, Rela	0.01
**Extracellular matrix organization**		
Non-integrin membrane-ECM interactions		
Syndecan interactions	Itgb1, Sdc2	0.04
**Hemostasis**		
Cell surface interactions at the vasculat wall		
Basigin interactions	Itgb1, cav1	0.01
**Immune System**		
Adaptive immune system		
Costimulation by the CD28 family		
CTLA4 inhibitory signalling	Src and Akt1	0.05
Innate Immune System		
DDX58/IFIH1-mediated induction of interferon-alpha/beta		
TRAF6 mediated NF-kB activation	App, Nfkb2, and Rela	0.02
Cytosolic sensors of pathogen-associated DNA		
ZBP1(DAI) mediated induction of type1 IFNs		
RIP-mediated NFkB activation via ZBP1	App, Nfkb2, and Rela	0.03
DEx/H-box helicases-activated type I IFN and inflammatory cytokines production	App, Nfkb2, and Rela	0.03
C-type lectin receptors (CLRs)		
CLEC7A(Dectin-1) signalling		
Dectin-1 mediated noncanonical NF-kB signalling	Psmd2, Nfkb2, Fbxw11, and Rela	0.03
Cytokine Signalling in Immune system		
Signalling by Interleukins		
Interleukin-1 family signalling		
Interleukin-1 processing	Nfkb2 and Rela	0.02
**Metabolism**		
Metabolism of lipids		
Triglyceride metabolism		
Triglyceride catabolism	Cav1, Prkacb, and Ppp1ca	0.04
Phospholipid metabolism		
PI Metabolism		
Synthesis of PIPs in the nucleus	Pip4k2b	0.02
**Signal Transduction**		
Signalling by receptor tyrosine kinases		
Signalling by NGF		
NGF signalling via TRKA from the plasma membrane		
Signalling to ERKs		
Signalling to RAS		
p38MAPK events	Mapk14 and Src	0.03
Signalling by hedgehog		
Hedgehog ‘on’ state		
GLI proteins bind promoters of Hh responsive genes to promote transcription	Gli1	0.04
MAPK family signalling cascades		
RAF/MAP kinase cascade		
MAP2K and MAPK activation	Src, Ywhab, and Vcl	0.03
Intracellular signalling by second messengers		
PIP3-activated AKT signalling		
Negative regulation of the PI3K/AKT network	Src, Akt1, Pip4k2b, and Egfr	0.05

## Discussion

MA abuse is a major health concern and a social problem worldwide, and the mechanism of addiction still remains unclear. Clinically, it is crucial to understand MA addiction because of a current lack of effective pharmacotherapy and a high recurrence rate^31^. Therefore, it is important to make an accurate diagnosis of MA abuse and addiction in clinical medicine. In fact, non-invasive diagnostic tools or reliable biomarkers used to determine MA abuse and addiction are being sought.

The purpose of the present study was to investigate the transcriptional changes caused by rewarding effects in MA self-administration rats. Drug addiction is not a static condition but a chronic relapsing disorder, which develops with the repetition of rewarding and withdrawal effects caused by temporary drug use and abstinence, respectively. Therefore, it is not enough to only study static changes of genes for MA addiction. The transcriptional gene changes caused by initial reward and withdrawal could affect the following stages and play a key role in MA addiction. To find diagnostic genes in this process, we examined dynamic changes in mRNA expression in the rat whisker follicle during MA self-administration. We used the short-access rat model of MA self-administration and measured the number of drug infusions that each rat chose (reward rate), and it was found that the rat showed an increased rewarding effect upon repeated MA administration. Analysis of RNA-seq data revealed that time-dependent changes of gene expression patterns at the three time-points (CON, MASA, and WD) were different. Notably, the majority of genes showed a similar pattern of changes in expression: an initial increase followed by a decrease during MASA and WD, suggesting that these genes may play a similar role in maintaining the reward state caused by MA.

Network analysis is a tool for gene prioritization and can facilitate a better understanding of network interconnections among interactive genes. Functional gene-gene association network analysis is an effective way to understand the interaction of a large number of genes^25^. The importance of genes can be determined by measuring BC scores in the network^32,33^. In this study, a topological centrality analysis was performed using a functional association network to narrow down the candidate genes related to MA addiction, and 43 genes were selected as core genes in the network.

The top three core genes were heat shock protein (HSP) 90-beta 1 (*Hsp90ab1)*, RAC-alpha serine/threonine-protein kinase (*Akt1)*, and *Src*. They have been previously reported as drug addiction-related genes^18,29,30^; therefore, it is possible that they play an important role in MA addiction. The gene with the highest BC score was *Hsp90ab1* (BC score, 0.2127), and *Hsp90-beta* protein, encoded by *Hsp90ab1* gene, is a molecular chaperone maintaining the functional stability and viability of cells^34^. The expression of *Hsp90ab1* in the rat frontal cortex increases after morphine self-administration^35^. Furthermore, *Akt1*, which had the second highest BC score (0.1694) in this study, is implicated in excessive alcohol drinking behaviours^36^. Src-family kinase activation is necessary for incentive, motivational, and/or memory processes that promote contextual cocaine-seeking behaviours^30^. To determine the biological function of 43 genes that could act as a functional core in the constructed gene network, we performed functional enrichment analysis using the Reactome database. Reactome categorises large quantities of genes and proteins into functions and pathways based on a massive database^37^. Using the Reactome database, we identified the main enriched pathways of the selected 43 genes and discovered eight functional groups: cell-cell communication, developmental biology, diseases, extracellular matrix organization, hemostasis, the immune system, metabolism, and signal transduction. Notably, a prominent increase in the expression of genes related with AD and heparan sulfate biosynthesis was found.

AD is a chronic neurodegenerative disease and the most common age-related dementia^38^. Previous studies suggest that MA abuse is related to long-term damage in the human brain^39^. In fact, clinical studies using proton magnetic resonance spectroscopy showed that the level of N-acetyl-aspartate, a neuronal marker, reduced by 5% in MA abusers, and the reduction was also observed in several brain diseases, such as AD, stroke, and epilepsy^39^. The representative characteristics of AD are progressive loss of cognitive abilities, severe neurodegeneration, and neuroinflammation^40^. One of the hallmarks of AD is the formation of neuritic plaques, which contain filamentous extracellular β-amyloid (Aβ) aggregates. Aβ is generated by sequential cleavage of APP by NF-κB-dependent activation of β- and γ-secretase^41^. A previous study revealed that MA enhanced Aβ accumulation through alpha7 nicotinic acetylcholine receptor in brain endothelial cells^42^. In agreement with the aforementioned results, an increased expression of NF-κB, APP, and Aβ was found in the whisker follicles in MA self-administered rats in this study.

Heparan sulfate biosynthesis is another interesting pathway in this study. Heparan sulfate proteoglycans (HSPGs) are macromolecules located in the cell surface and extracellular matrix. HSPGs consist of core protein-linked glycosaminoglycan chains^43^. The core proteins of HSPGs are agrin, perlecan, glypicans, and syndecans (*Sdc*). In this study, a significant increase in the expression of *Sdc2* was found at the MASA session. In addition, most of genes included in the heparan sulfate pathway showed the same expression pattern as did *Sdc2*. According to previous studies, *Sdc* has four categories of functions: (1) growth factor receptor activation^44^; (2) matrix adhesion^45^; (3) cell to cell adhesion^46^; and (4) tumour suppression and progression^47^. *Sdc2* is involved in synaptic formation^48^, and *Sdc3* has been identified as a resilience factor of cocaine addiction^49^. Consequently, transcriptional alteration of *Sdc2* at the MASA session may be an important marker of MA addiction.

Drug addiction is a complicated, chronic disorder and is conceptualised as a cycle comprising rewarding effect, withdrawal effect, craving, and relapse to drug seeking behaviour^50,51^. Briefly, intake of drugs initially causes rewarding effect, and tolerance and withdrawal effects gradually occur after repeated use of the drugs. To escape the effects, craving to the drug intake arises, leading to relapse of drug-seeking behaviour. Therefore, a validated model of intravenous drug self-administration relevant to the transition to addiction is needed to uncover the complicated clinical situation in human addicts. In the present study, rats were allowed to self-administer MA 2 h daily for 16 days to determine the mechanism underlying the transition from initial drug use to drug addiction. However, the present study has some limitations. For example, a short access rat model of MA self-administration was used in this study. Recent studies have shown that rats with extended access to MA, compared with rats with short access to MA, showed enhanced MA-primed reinstatement of drug-seeking and cognitive deficits^52–55^. Moreover, extended limited daily access to MA self-administration in animal models mimics the course of MA administration in human^55^. Therefore, the model in the present study can be used to study the reward process, which is an initial step of the MA addiction. However, a follow-up study with long access self-administration would be needed to understand MA addiction in human.

In conclusion, the present findings suggest that the alteration of the gene expression and signalling networks in the whisker follicles in MA self-administered rats can be used to understand the biological changes and MA rewarding effects induced by MA self-administration. Further studies should be needed to determine the differential changes in gene expression between the whisker follicle and the brain to strengthen our conclusion.

## Methods

### Animals

Adult male Sprague-Dawley rats (Daehan Animal, Seoul, Republic of Korea), 310–350 g in weight, were housed individually in the laboratory animal facility in room temperature (22 ± 2 °C) and humidity (60 ± 2%) controlled cages on a 12 h light/dark cycle (lights on at 7:00 AM). Animals had access to food and water *ad libitum*. All experimental procedures were approved by the institutional animal care and use committee at Daegu Hanny University, Daegu, Republic of Korea (DHUMC-D-13002-PRO-02, 2013). The experiments were carried out in accordance with the university’s scientific research guidelines and regulations.

### MA self-administration and sample collection

MA self-administration was conducted as previously described^56^. Briefly, rats were habituated to their environment for 1 week, and they were then implanted with chronic indwelling jugular catheters and assigned to the control (n = 6) or MA self-administration (n = 6) group. One week after surgery, rats were given food training. Subsequently, they were treated with a FR1 schedule of MA self-administration with 2 h access per day to MA hydrochloride (0.05 mg·kg·infusion^−2^) dissolved in sterile heparinised saline (3 U·mL^−1^) for 2 weeks. If animals had shown the stable infusion numbers for 3 consecutive days (variation less than 10% of the mean infusion number of the 3 days), the mean infusion number was regarded as the baseline. Saline self-administered rats received heparinised saline infusions during the self-administration period. All responses were recorded using MED associated software. Samples were collected 3 times during the experiment: Control (CON) samples were collected before MASA; MASA samples were collected within 2 hours after the last MA self-administration; and WD samples were collected 1 month after MASA.

### RNA isolation

Whisker samples were collected and stored at −80 °C before RNA extraction after rats were sacrificed. Total RNA was isolated from the whisker roots (hair follicles) using the spin column type RNA extraction kit (Bioneer, Republic of Korea). The quality and concentration of RNA were measured using a filter-based multi-mode microplate reader (FLUOstar Omega, BMG LABTECH, Germany). Residual genomic DNA was removed by incubation with RNase-free DNase I for 30 min at 37 °C.

### Construction of transcriptome libraries

The mRNA sequencing libraries were prepared according to the Illumina TruSeq RNA sample preparation kit v2 (Illumina, Inc., San Diego, CA, USA). Briefly, 1 μg total RNA was used for polyA mRNA selection using streptavidin-coated magnetic beads, followed by thermal fragmentation of selected mRNA. The 200–500 bp fragmented mRNA was used as a template for cDNA synthesis by reverse transcriptase with random primers. The cDNA was converted into double-stranded DNA that was end-repaired (to incorporate the specific index adaptors for multiplexing), purified, and amplified for 15 cycles. The quality and functionality of the final amplified libraries were examined with a DNA high sensitivity chip on an Agilent 2100 Bioanalyzer (Agilent Technologies, Palo Alto, CA, USA). Libraries were prepared according to the Illumina’s protocol. Briefly, random fragmentation of the DNA or cDNA, followed by 5′ and 3′ adapter ligation, was conducted. Alternatively, “tagmentation” combined the fragmentation and ligation reactions into a single step, which greatly increased the efficiency of the library preparation process. Subsequently, adapter-ligated fragments were PCR amplified and gel-purified. The concentration of each library was measured by real-time PCR. Agilent 2100 Bioanalyzer was used for profiling the distribution of insert size. For cluster generation, the library was loaded into a flow cell where fragments were captured on a lawn of surface-bound oligos complementary to the library adapters. Each fragment was then amplified into distinct clonal clusters through bridge amplification. After cluster generation was completed, the clustered libraries were sequenced by Illumina HiSeqTM 2500 according to the manufacturer’s instructions (Illumina Inc., USA), and paired-end sequencing was performed for 100 cycles using TruSeq rapid SBS kit or TruSeq SBS kit v4. The sequencing protocol, the HiSeq^TM^ 2500 system user guide part #15011190 Rev. V HCS 2.2.58, was used in this study.

### Sequencing data analysis

Sequencing raw images were generated using HiSeqTM Control Software (HCS v2.2), and base calling was performed through an integrated primary analysis software called RTA (v1.18). Images generated by HiSeqTM2500 were converted into nucleotide sequences by base calling and were stored in the FASTQ format using the Illumina package bcl2fastq (v1.8.4). Clean reads were generated by filtering the dirty reads, which contained adaptors and unknown or low phred quality scored-bases, from raw reads. Clean reads were mapped to reference Rattus norvegicus genome (rn6) and gene sequences using a conventional Tuxedo protocol. Reading mismatches of ≤5 bases were allowed in the alignment. Reads that matched with reference rRNA sequences were also mapped and removed. Tophat2 software and the Cufflinks package (v2.2.1) were used to calculate FPKM (Fragments per kb per million reads) values for each gene used for subsequent data analysis. DEGs were analysed using one-way ANOVA, Student’s *t*-test (p < 0.05), and fold changes. Hierarchical clustering and PCA were also performed with the GeneSpring 7.3 software (Agilent Technologies).

### Gene expression pattern analysis

Transcripts that were differentially expressed between the groups were analysed using one-way ANOVA. After primary data alignment and further filtration to find transcripts with >1.1 or <0.9 fold-change between groups, 1,890 transcripts were quantified, and 666 genes were next identified with same patterns in both MASA/CON and WD/MASA.

### Functional association network analysis

The STRING-database (ver. 10.0) was used to conduct a rat gene functional association network using physical and functional link information extracted *via* the retrieval of interacting with a confidence score over 400. Network visualization and calculation of BC^32,33^ were performed using Cytoscape 2.8^57^.

### Statistical analysis

Data were analysed using a two-way ANOVA followed by a Student Newman-Keuls test in the self-administration experiment. Data from the self-administration group were compared with those from the saline self-administration group, and *p < 0.05, **p < 0.01, or ***p < 0.001 was considered statistical significance. The differences in the RNA-seq results within groups were evaluated by PCA. Differentially expressed transcripts between the groups were calculated using a one-way ANOVA. The Mann-Whitney U test was performed to compare links in the network, and the hypergeometric test was used for the comparison of relative fold-enrichment in the network.

## References

[1] Nestler EJ (2013). Cellular basis of memory for addiction. Dialogues in clinical neuroscience.

[2] Ross S, Peselow E (2009). The neurobiology of addictive disorders. Clinical neuropharmacology.

[3] Fillmore MT (2003). Drug abuse as a problem of impaired control: current approaches and findings. Behavioral and cognitive neuroscience reviews.

[4] Scott JC (2007). Neurocognitive effects of methamphetamine: a critical review and meta-analysis. Neuropsychol Rev.

[5] Larsen KE, Fon EA, Hastings TG, Edwards RH, Sulzer D (2002). Methamphetamine-induced degeneration of dopaminergic neurons involves autophagy and upregulation of dopamine synthesis. J Neurosci.

[6] Wen D (2016). Cholecystokinin-8 inhibits methamphetamine-induced neurotoxicity via an anti-oxidative stress pathway. Neurotoxicology.

[7] Warren MW (2007). Calpain and caspase proteolytic markers co-localize with rat cortical neurons after exposure to methamphetamine and MDMA. Acta Neuropathol.

[8] Smith KJ, Butler TR, Self RL, Braden BB, Prendergast MA (2008). Potentiation of N-methyl-D-aspartate receptor-mediated neuronal injury during methamphetamine withdrawal *in vitro* requires co-activation of IP3 receptors. Brain Res.

[9] Kalechstein AD, Newton TF, Green M (2003). Methamphetamine dependence is associated with neurocognitive impairment in the initial phases of abstinence. J Neuropsychiatry Clin Neurosci.

[10] Degenhardt L, Hall W (2012). Extent of illicit drug use and dependence, and their contribution to the global burden of disease. Lancet (London, England).

[11] United Nations Office on Drugs and Crime (UNODC), *2017*. *World Drug Report* 2017, http://www.unodc.org/wdr2017/field/4.1_Treatment.xlsx (2017).

[12] Owen GT, Burton AW, Schade CM, Passik S (2012). Urine drug testing: current recommendations and best practices. Pain Physician.

[13] Frederick DL (2012). Toxicology testing in alternative specimen matrices. Clinics in laboratory medicine.

[14] Lee S (2011). Analysis of pubic hair as an alternative specimen to scalp hair: a contamination issue. Forensic Sci Int.

[15] Maekawa M (2015). Utility of Scalp Hair Follicles as a Novel Source of Biomarker Genes for Psychiatric Illnesses. Biological psychiatry.

[16] Arck, P. C. *et al*. Stress inhibits hair growth in mice by induction of premature catagen development and deleterious perifollicular inflammatory events via neuropeptide substance P-dependent pathways. *The American journal of pathology* **162**, 803–814, S0002-9440(10)63877-1 (2003). 10.1016/S0002-9440(10)63877-1 PMC1868104 12598315

[17] Zhang J (2014). Transcriptional profiling in rat hair follicles following simulated Blast insult: a new diagnostic tool for traumatic brain injury. PloS one.

[18] Bolanos CA, Nestler EJ (2004). Neurotrophic mechanisms in drug addiction. Neuromolecular medicine.

[19] Koob, G. F. & Le Moal, M. Drug addiction, dysregulation of reward, and allostasis. *Neuropsychopharmacology: official publication of the American College of Neuropsychopharmacolog*y **2**4, 97–129, S0893-133X(00)00195-0 (2001). 10.1016/S0893-133X(00)00195-0 11120394

[20] Nestler, E. J. Molecular mechanisms of drug addiction. *Neuropharmacology* **47****1**, 24–32, S0028390804001911 (2004). 10.1016/j.neuropharm.2004.06.031 15464123

[21] Sharma D, Kim MS, D’Mello SR (2015). Transcriptome profiling of expression changes during neuronal death by RNA-Seq. Experimental biology and medicine (Maywood, N.J.).

[22] Zhu L (2016). mRNA changes in nucleus accumbens related to methamphetamine addiction in mice. Scientific reports.

[23] Sivachenko, A. Y., Yuryev, A., Daraselia, N. & Mazo, I. Molecular networks in microarray analysis. *Journal of bioinformatics and computational biology* **5**, 429–456, S0219720007002795 (2007). 10.1142/s0219720007002795 17636854

[24] Sharan, R. & Ideker, T. Modeling cellular machinery through biological network comparison. *Nature biotechnolog*y **2**4, 427–433, nbt1196 (2006). 10.1038/nbt1196 16601728

[25] Breen MS (2016). Candidate gene networks and blood biomarkers of methamphetamine-associated psychosis: an integrative RNA-sequencing report. Translational psychiatry.

[26] Proulx, S. R., Promislow, D. E. & Phillips, P. C. Network thinking in ecology and evolution. *Trends in ecology & evolution* **2**0, 345–353, S0169-5347(05)00088-1 (2005). 10.1016/j.tree.2005.04.004 16701391

[27] Huh WK (2003). Global analysis of protein localization in budding yeast. Nature.

[28] Jeon J (2011). Network clustering revealed the systemic alterations of mitochondrial protein expression. PLoS computational biology.

[29] Chen JC, Chen PC, Chiang YC (2009). Molecular mechanisms of psychostimulant addiction. Chang Gung medical journal.

[30] Xie X, Arguello AA, Wells AM, Reittinger AM, Fuchs RA (2013). Role of a hippocampal SRC-family kinase-mediated glutamatergic mechanism in drug context-induced cocaine seeking. Neuropsychopharmacology.

[31] Brackins T, Brahm NC, Kissack JC (2011). Treatments for methamphetamine abuse: a literature review for the clinician. Journal of pharmacy practice.

[32] Freeman LC (1978). Centrality in social networks conceptual clarification. Social Networks.

[33] Riquelme Medina I, Lubovac-Pilav Z (2016). Gene Co-Expression Network Analysis for Identifying Modules and Functionally Enriched Pathways in Type 1 Diabetes. PloS one.

[34] Chiosis G (2006). Targeting chaperones in transformed systems–a focus on Hsp90 and cancer. Expert opinion on therapeutic targets.

[35] Koshimizu TA (2010). Inhibition of heat shock protein 90 attenuates adenylate cyclase sensitization after chronic morphine treatment. Biochemical and biophysical research communications.

[36] Neasta J, Ben Hamida S, Yowell QV, Carnicella S, Ron D (2011). AKT signaling pathway in the nucleus accumbens mediates excessive alcohol drinking behaviors. Biological psychiatry.

[37] Croft D (2014). The Reactome pathway knowledgebase. Nucleic acids research.

[38] Ferri, C. P. *et al*. Global prevalence of dementia: a Delphi consensus study. *Lancet (London, England)* **36**6, 2112–2117, S0140-6736(05)67889-0 (2005). 10.1016/S0140-6736(05)67889-0 PMC2850264 16360788

[39] Ernst T, Chang L, Leonido-Yee M, Speck O (2000). Evidence for long-term neurotoxicity associated with methamphetamine abuse: A 1H MRS study. Neurology.

[40] Serrano-Pozo A, Frosch MP, Masliah E, Hyman BT (2011). Neuropathological alterations in Alzheimer disease. Cold Spring Harbor perspectives in medicine.

[41] Guo Q, Robinson N, Mattson MP (1998). Secreted beta-amyloid precursor protein counteracts the proapoptotic action of mutant presenilin-1 by activation of NF-kappaB and stabilization of calcium homeostasis. J Biol Chem.

[42] Liu L (2017). Alpha7 nicotinic acetylcholine receptor is required for amyloid pathology in brain endothelial cells induced by Glycoprotein 120, methamphetamine and nicotine. Sci Rep.

[43] Christianson HC, Belting M (2014). Heparan sulfate proteoglycan as a cell-surface endocytosis receptor. Matrix biology: journal of the International Society for Matrix Biology.

[44] Ma, P. *et al*. Heparanase deglycanation of syndecan-1 is required for binding of the epithelial-restricted prosecretory mitogen lacritin. *The Journal of cell biology* **174**, 1097–1106, jcb.200511134 (2006). 10.1083/jcb.200511134 PMC1666580 16982797

[45] Bernfield M (1992). Biology of the syndecans: a family of transmembrane heparan sulfate proteoglycans. Annual Review of Cell Biology.

[46] Carey DJ (1997). Syndecans: multifunctional cell-surface co-receptors. The Biochemical journal.

[47] Choi, S. *et al*. Transmembrane domain-induced oligomerization is crucial for the functions of syndecan-2 and syndecan-4. *The Journal of biological chemistry* **280**, 42573–42579, M509238200 (2005). 10.1074/jbc.M509238200 16253987

[48] Hu HT, Umemori H, Hsueh YP (2016). Postsynaptic SDC2 induces transsynaptic signaling via FGF22 for bidirectional synaptic formation. Scientific reports.

[49] Chen J (2013). Hypothalamic proteoglycan syndecan-3 is a novel cocaine addiction resilience factor. Nature communications.

[50] Cami J, Farre M (2003). Drug addiction. The New England journal of medicine.

[51] Goldstein RZ, Volkow ND (2002). Drug addiction and its underlying neurobiological basis: neuroimaging evidence for the involvement of the frontal cortex. Am J Psychiatry.

[52] Edwards S, Koob GF (2013). Escalation of drug self-administration as a hallmark of persistent addiction liability. Behav Pharmacol.

[53] Mandyam CD (2008). Varied access to intravenous methamphetamine self-administration differentially alters adult hippocampal neurogenesis. Biological psychiatry.

[54] Recinto P (2012). Levels of neural progenitors in the hippocampus predict memory impairment and relapse to drug seeking as a function of excessive methamphetamine self-administration. Neuropsychopharmacology.

[55] Schwendt M (2009). Extended methamphetamine self-administration in rats results in a selective reduction of dopamine transporter levels in the prefrontal cortex and dorsal striatum not accompanied by marked monoaminergic depletion. J Pharmacol Exp Ther.

[56] Yoon SS (2010). Acupuncture suppresses morphine self-administration through the GABA receptors. Brain research bulletin.

[57] Cline MS (2007). Integration of biological networks and gene expression data using Cytoscape. Nature protocols.

